# Report on the first SLFN11 monothematic workshop: from function to role as a biomarker in cancer

**DOI:** 10.1186/s12967-017-1296-3

**Published:** 2017-10-02

**Authors:** Alberto Ballestrero, Davide Bedognetti, Domenico Ferraioli, Paola Franceschelli, Sana Intidhar Labidi-Galy, Elisabetta Leo, Junko Murai, Yves Pommier, Petros Tsantoulis, Valerio Gaetano Vellone, Gabriele Zoppoli

**Affiliations:** 1 Istituto di Ricovero e Cura a Carattere Scientifico, Policlinico San Martino IST-Istituto Nazionale Tumori, Genoa, Italy; 2 Università degli Studi di Genova, Viale Benedetto XV, 6, 16132 Genoa, Italy; 3 Tumor Biology Immunology and Therapy Branch, Sidra Medical and Research Center, Doah, Qatar; 4 Centre de Lutte contre le Cancer Léon Bérard, Lyon, France; 5 Department of Oncology, Hôpitaux Universitaires de Genève, Geneva, Switzerland; 6 Oncology, Innovative Medicine Early Development, AstraZeneca, Cambridge, UK; 7 Laboratory of Molecular Pharmacology, Center for Cancer Research, National Cancer Institute, National Institutes of Health, Bethesda, MD USA

**Keywords:** SLFN11, Biomarker, Immune system, DNA damage repair, Chemotherapy, Prognosis, Prediction, Ovarian cancer, Colorectal cancer, Breast cancer

## Abstract

SLFN11 is a recently discovered protein with a putative DNA/RNA helicase function. First identified in association with the maturation of thymocytes, SLFN11 was later causally associated, by two independent groups, with the resistance to DNA damaging agents such as topoisomerase I and II inhibitors, platinum compounds, and other alkylators, making it an attractive molecule for biomarker development. Later, SLFN11 was linked to antiviral response in human cells and interferon production, establishing a potential bond between immunity and chemotherapy. Recently, we demonstrated the potential role of SLN11 as a biomarker to predict sensitivity to the carboplatin/taxol combination in ovarian cancer. The present manuscript reports on the first international monothematic workshop on SLFN11. Several researchers from around the world, directly and actively involved in the discovery, functional characterization, and study of SLFN11 for its biomarker and medicinal properties gathered to share their views on the current knowledge advances concerning SLFN11. The aim of the manuscript is to summarize the authors’ interventions and the main take-home messages resulting from the workshop.

## Introduction: SLFN11 as a potential predictive biomarker to assess response to DNA damage inhibitors

### Presented by Gabriele Zoppoli

**Fig. 1:**
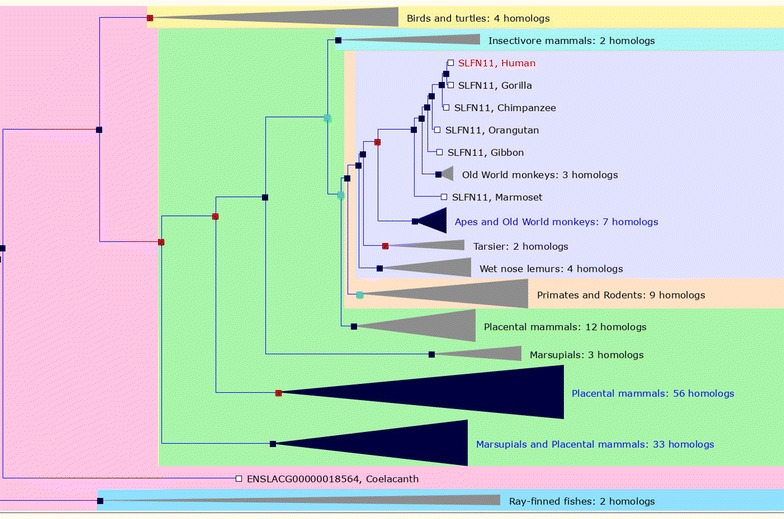
Conservation tree of SLFN11 across species. Constructed using the Ensembl! GeneTree tool, queried with the term “SLFN11” (last accessed 2017, June 19)

SLFN11 is a putative DNA/RNA helicase, first described for its role in thymocyte development and differentiation in mouse models [1]. SLFN11 is part of a family of proteins with various degree of homology across species, but intriguingly being consistently present only in vertebrates and especially in mammals (Fig. 1). The helicase domain is present only in the “long” SLFN proteins such as SLFN11, whereas the “short” SLFN proteins share only a domain of unknown function (the SLFN domain); finally, the intermediate SLFN proteins also possess a highly conserved SWADL motif, but lack the helicase domain [2]. Recently, while correlating the in vitro activity of topoisomerase I (TOP1) inhibitors with the transcriptional profiles of more than 20,000 genes in the NCI-60 cancer cell line model, we discovered by serendipity an unusually strict association between the levels of SLFN11 and the sensitivity to such DNA damaging agents (DDA). Subsequently, we observed that such high correlation was maintained with TOP2 inhibitors, as well as alkylating agents such as cisplatin. We then corroborated our discovery by modulation of SLFN11 expression in lung, colon, breast, and prostate cancer cell lines (HOP-62, HCT-116, MDA-MB-231, and DU-145 respectively), thereby demonstrating the causal relationship between SLFN11 intracellular levels and sensitivity to DDA [3]. Independently, Barretina and co-authors reported that Ewing’s sarcoma cell lines had very high SLFN11. In line with our findings, those authors also described the tight correlation between SLFN11 transcript expression and TOP1 inhibitor toxicity in cancer cells [4]. In parallel with the findings concerning SLFN11 in cancer, its role and relation with the immune system, as well as the property of behaving as an early interferon-response gene were described [5]. Taken together, the published data points toward a possible connection between SLFN11, immunity and cancer. Indeed, it was not long since scientific reports appeared, describing SLFN11 as a biomarker of response to DDA in human cancer. Moreover, since no evident mutations or copy number variations of SLFN11 could be find in cell models or in patients’ cohorts such as the cancer genome atlas (TCGA), researchers have focused their attention on SLFN11 regulation by methylation. Indeed, SLFN11 hypermethylation is associated with worse prognosis in ovarian cancer and with a poor response to platinum derived compounds in lung cancer [6]; consistently we have observed that SLFN11 overexpression purports a platinum-sensitive phenotype in patients affected by such neoplasm. More recently, SLFN11 has been associated with sensitivity to PARP inhibitors and other DDA in both cancer models and in clinical case sets. In conclusion, SLFN11 appears as a promising molecule both for its causative implication in sensitivity to DDA, as a biomarker of response to such agents, and for its potential as a link between immunity, cancer, and response to chemotherapy.

## Cell cycle inhibitory function of SLFN11 in the DNA damage response (DDR)

### Presented by Elisabetta Leo

SLFN11 was recently identified as a novel DNA damage response (DDR) gene in cancer cell genomic analyses of the NCI60 [3] and the cancer cell line encyclopedia (CCLE) [4] cancer cell models. In 2012 we reported the causative effect of SLFN11 as a determinant of cancer cell sensitivity to multiple DNA damaging agents in different human cell lines: upon downregulation of SLFN11 by siRNA, cells showed a dramatic increase in viability after short and long-term treatments with camptothecin (CPT) and other DNA damaging agents (DDA) when compared to SLFN11-proficient cell lines [3]. After these initial observations, we worked to elucidate the mechanisms by which SLFN11 impinges on the DDR.

We found that, in SLFN11 proficient cells, SLFN11 protein levels are constant in all the phases of the cell cycle (after FACS-sorting as well as upon pharmacological synchronization) G1, S, G2 and mitosis. Subcellular fractionation studies revealed that SLFN11 is preferentially localized in the nuclear compartment, and binds tighter to the chromatin upon accumulation of DNA damage. SLFN11 in the nucleus forms foci that are in close proximity ahead of the replication foci. SLFN11 is preferentially present in the euchromatic regions (open chromatin, identified by H3K9Ac) and is clearly excluded from heterochromatin (H3K9Me3). We also examined cell cycle progression and DNA replication after CPT treatment in SLFN11-proficient versus SLFN11-downregulated cells. In non-treated cells there is no apparent phenotypic difference; however, during treatment with low doses of CPT, dramatic differences in cell cycle and in DDR are observed between SLFN11-proficient and -deficient cells; these differences are visible as early as 4 h after DDA-treatments [7]. If cells are SLFN11 proficient, they undergo an enforced G1/S arrest, with tight cell cycle and replication block, which leads to cell death [8, 9]. On the contrary, if SLFN11 is absent, cells are capable to re-enter the cell cycle, slowly progress through S-phase and are less prone to die. This slow progression is associated to an hyper-activation of the DNA replication and damage checkpoint: indeed, very high and persistent phosphorylation of ATM, ATR, Chk1, an Chk2 are observed. When SLFN11-depleted cells are co-treated with CPT and either ATM, ATR or Chk1/2 inhibitors, they progress much faster through S-phase and they are ultimately re-sensitized to the damage.

We suggest that SLFN11 works as an additional cell cycle checkpoint, that possibly acts upstream of the classical replication and damage checkpoint, preventing the cells to progress and to survive when they accumulate DNA damage and replication stress [7]. Based on these observations, we can conclude that SLFN11 has high potential relevance in the clinics as predictive biomarker for patient stratification. SLFN11-proficient tumors may be more likely to respond to a DDA-based chemotherapy, whereas SLFN11-deficient tumors might require more aggressive combination treatments, for example with ATM or ATR inhibitors, or different anticancer strategies.

## SLFN11 induces lethal S-phase arrest in response to DNA damage—a novel mechanism of how cancer cells are killed by DNA damaging agents

### Presented by Yves Pommier and Junko Murai

**Fig. 2:**
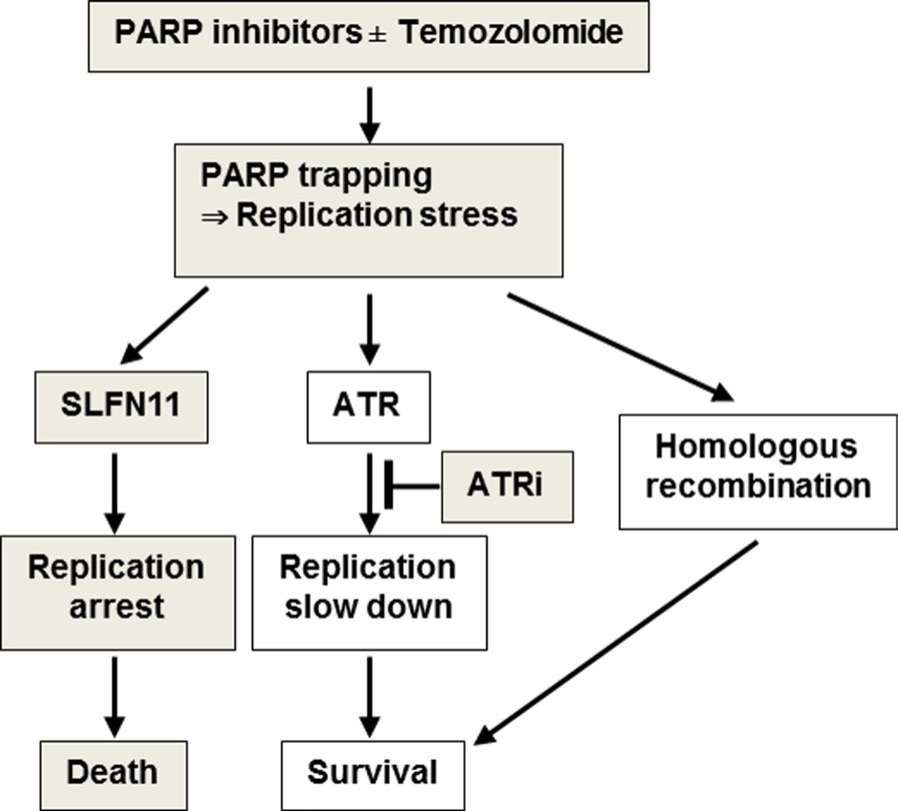
Summary scheme proposing the role of SLFN11 in parallel to ATR and homologous recombination [12]

In two works previously published in 2012 and 2014, our group reported the founding of a novel mechanism of action for PARP inhibitors named PARP-trapping, which explains why PARP inhibitors act as DNA damaging agents [10, 11]. PARP inhibitors trap PARP1 and PARP2 at DNA single strand break lesions, which are common and highly cytotoxic because of inducing replication stress. The potency of PARP trapping is widely different among clinical PARP inhibitors, and talazoparib is the most potent PARP trapping inhibitor so far. We reported that sensitivity profile of talazoparib in NCI-60 is highly correlated with the expression profile of SLFN11. The correlation was shown to be causal using four isogenic cell lines (parental cells with high SLFN11 expression vs their SLFN11-knockout cells) and extended to other PARP inhibitors including olaparib, and the combination of talazoparib and temozolomide. Although deficiency of homologous recombination is a dominant determinant of hypersensitivity to PARP inhibitors, SLFN11 sensitized cells in a parallel pathway with homologous recombination deficiency. SLFN11 induced irreversible and lethal S-phase arrest under continuous talazoparib treatment for 48 h, while cells without SLFN11 slowly reached G2-phase and viable at that time under the regulation of S-phase checkpoint by ATR activation. The abrogation of S-phase checkpoint by the addition of ATR inhibitor (ATRi) with PARP inhibitors, which enforces unscheduled origin firing, synergized cells drastically. Hence, we propose two distinct strategies to kill cancer cells (Fig. 2) using PARP inhibitors; one is to induce SLFN11-dependent replication arrest by PARP inhibitor alone or the combination with temozolomide, the other is to use PARP inhibitors with ATR inhibitors to induce lethal unscheduled origin firing in SLFN11-deficient cells [12].

## Predictive markers in ovarian cancer

### Presented by Domenico Ferraioli

Ovarian cancer is the seventh most common cancer worldwide and the eighth cause of cancer death in women [13]. Early stages are hard to detect, and several patients are diagnosed when the disease is already in an advanced stage [14]. Standard recommendations for patients with advanced ovarian cancer (AOC) include primary debulking surgery (PDS) followed by platinum-based adjuvant chemotherapy [15] but, in some cases, the PDS is not feasible or is associated with unacceptable morbidity; therefore, neoadjuvant chemotherapy (NACT) followed by debulking surgery should be performed [16]. Patient response to chemotherapy for ovarian cancer is extremely heterogeneous and approximately 60% of patients with AOC will relapse after first-line chemotherapy [17]. Nowadays, tools predicting the sensitivity or the resistance to chemotherapy and allowing treatment stratification are not available; nevertheless, different biomarker assays are in active development. These approaches include functional assays, identification of resistance gene markers, and micro RNA analysis. A systematic review of 42 studies concerning the prediction of chemotherapy response in AOC using gene expression was performed in 2015 by Lloyd et al. [18]. The authors concluded that a clinically applicable gene signature cannot be identified, highlighting the presence of a severe heterogeneity concerning the histological type, the tissue preservation techniques applied, and the manners of obtaining the gene signature among the different studies. Chemoresponse tests, or other biomarker assays, are thus not recommended to choose a chemotherapy regimen. The majority of the available studies failed to demonstrate a survival benefit of chemotherapy regimens selected on chemoresponse assays compared to chemotherapy regimens selected using traditional clinical factors [15]. To conclude, a validated predictive biomarker does not currently exist, and the international guidelines only suggest the use of CA 125 to monitor response to chemotherapy as part of a clinical trial [19]. Well-designed randomized controlled trials are needed to develop a predictive model of response to chemotherapy.

## SLFN11 assessment in ovarian cancer: phenotypic and histological distribution and association with TIL infiltration

### Presented by Valerio Gaetano Vellone

Epithelial carcinoma of the ovary has always been clinically considered as one disease, but there is now a much greater realization that the various subtypes have a different natural behavior and prognosis [20]. At present, adjuvant therapy is mainly dependent upon tumor stage and grade rather than type [15]. However, it is of common observation how tumors with similar stage and histologic type can behave in radical different ways and finding potential molecular markers represents one of the challenges of modern surgical pathology. To date, DNA-damaging chemotherapeutic agents constitute the backbone of treatment for most solid and hematological tumors. High expression levels of SLFN11 seems to correlate with the sensitivity of human cancer cells to DNA-damaging agents [3]. In this setting, it appears clear how immunohistochemistry (IHC) testing for SLFN11 may represent a powerful tool to predict the response and modulate the chemotherapy for high-grade serous ovarian carcinoma (HGSC). To date no commercial kit for SLFN11 IHC testing is available, so we adapted two kits originally commercialized for Western Blot (WB), and we tested a population of 75 cases of HGSC. As positive control, we used a commercial culture of ovarian carcinoma (SKOV-3) processed with agarose-embedded cell block technique (CCB); SKOV-3 cell culture is known to have high level of expression for SLFN11.

**Fig. 3:**
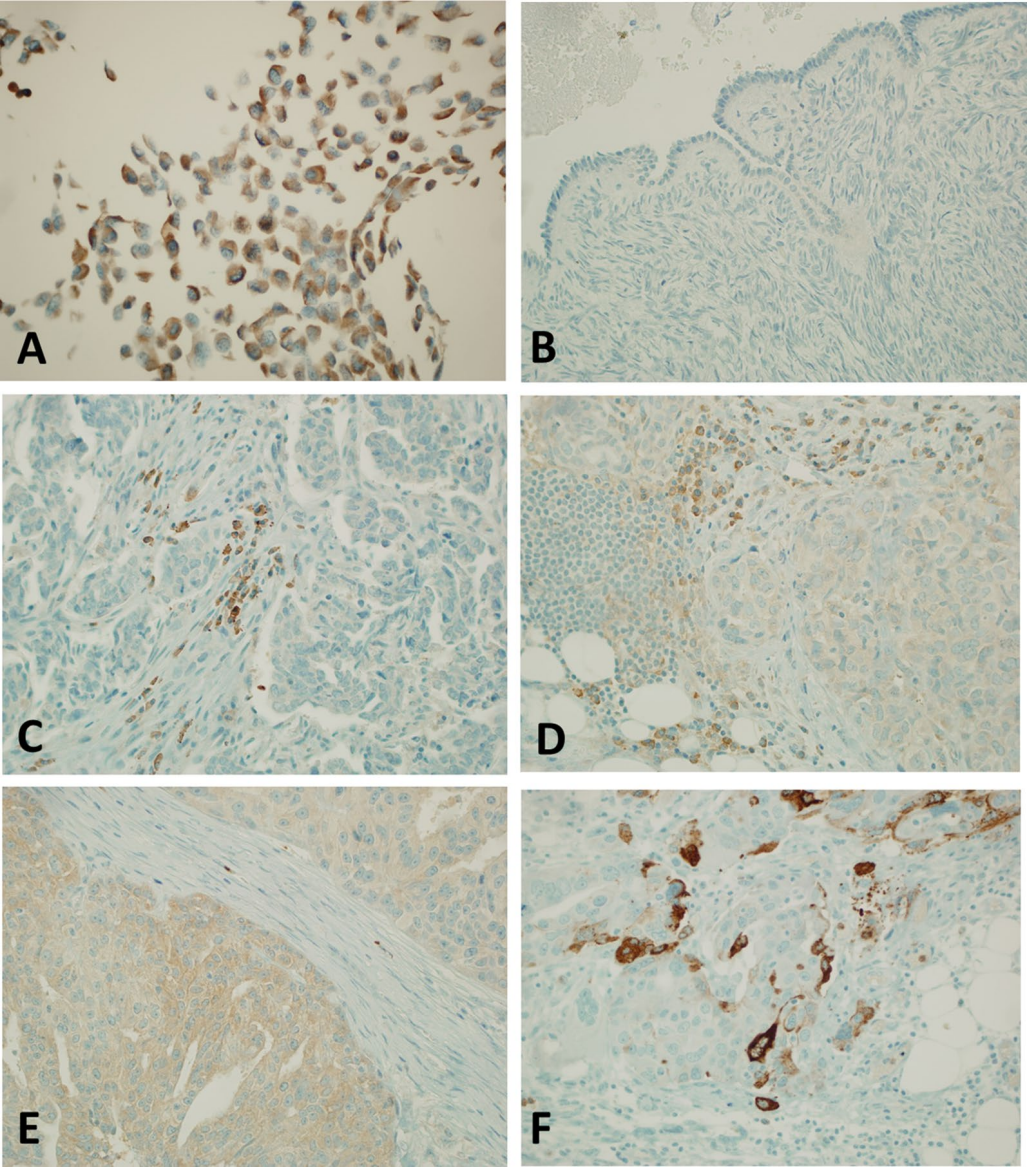
Immunohistochemistry staining for SLFN11: **A** (IHC; 400×) Positive external control constituted by SKOV-3 cell block culture. **B** (IHC; 400×) Negative external control constituded by normal menopausal ovary: no stain in both overian surface cells and stromal cells. **C** (IHC, 400×) SLFN negative HGSC: cancer cells show no stain, TILs show an intense stain representing a useful internal control.** D** (IHC, 400×) SLFN low HGSC (HS 2) with a faint (IS 1 +) inconstant (DS 2 +) pattern of stain. **E** (IHC, 400×) SLFN intermediate HGSC (HS4) with a moderate (IS 2 +) inconstant (DS 2 +) pattern of stain. **F** (IHC, 400×) SLFN high HGSC (HS 6) with an intense (IS 3 +) but inconstant (DS 2 +) pattern of stain

In some cells we observed a crescent-shaped thickening of the coloration in the perinuclear area consistent with Golgi complex (Fig. 3A). No staining was observed in the nuclei. This observation is in apparent contrast with what was reported by Zoppoli et al. [3] and by Dr E. Leo in their work. However, nuclear antigens may translocate into cytoplasm or dispersed by nuclear wall disruption upon either apoptonecrotic processes intervening during tissue exeresis or due to the fixation processes. Hence, we cannot currently conclude that SLFN11 staining in formalin fixed, paraffin-embedded cells reflects SLFN11 location in living tissues. It has to be noted that, although Dr Leo’s subcellular fractionation showed a preferential nuclear localisation, a proportion of SLFN11 was also present in the cytoplasm. Furthermore, at least two publications have pointed out that SLFN11 may be found also in cytoplasm, so currently we can only speculate that this protein may translocate following active processes in living/dying cells as well [1, 21].

Immunohistochemistry appeared clean and specific, no aspecific bonds were observed in tumor and residual ovarian stroma (Fig. 3B), and a relevant positive internal control was represented by a subpopulation of tumor infiltrating lymphocytes (TIL), which stained intensively for SLFN11 (Fig. 3C). SLFN11 expression resulted extremely variable among cases, and even in different fields of the same tumor. However, a dominant pattern of intensity seems to exist in the same neoplasia. For each case we assessed both the intensity score (IS) and the distribution score (DS) evaluating at least 300 cells. Intensity score (IS) evaluates the main pattern of intensity of stain in positive cancer cells as follow: 0: no stain (Fig. 3C); 1 ±: weak stain (visible at high magnification) (Fig. 3D); 2 +: moderate stain (visible at scan magnification) (Fig. 3E); 3 +: intense stain (Fig. 3F). Distribution score (DS) evaluates the percentage of stained cancer cells as follow: 0: no stained cells; 1 +: < 10% of stained cells; 2 +: 10–40% of stained cells; 3 +: > 40% of stained cells. These scores were combined to obtain a final histological score (HS) as follow: HS = IS × DS. Study cases were grouped on the base of HS in the following categories: cases with HS = 0 were considered SFLN11 negative, cases with HS 1 and 2 were considered SFLN11 low, cases with HS 3 and 4 were considered SFLN11 intermediate, while cases with HS 6 and 9 were considered SFLN11 high. At the end of the evaluation, the SLFN11 expression in the studied case set was distributed with an elegant Gaussian-like fashion: 27 cases (39.13%) resulted SLFN11 negative, 11 cases (15.94%) resulted SLFN11 low, 23 cases (33.33%) resulted SLFN11 intermediate and 8 cases (11.59%) resulted SLFN11 high. Globally, SLFN11 appears to be poorly expressed in HGSC, with the larger subpopulation composed by cases with no sign of stain (SLFN11 negative). We hypothesize that, if SLFN11 negative cases mirror the large population of chemotherapy-resistant patients, SLFN11-high cases may identify a subpopulation of chemotherapy-responsive patients with a better prognosis. Of interest, only a subpopulation of TIL appears to express SLFN11. Their nature and biological role remain to be studied. In the future the presented IHC data will be matched with RNA expression data and clinical data such as overall survival and disease free interval, to better estimate the role of SLFN11 as a potential novel, pivotal prognostic marker in HGSC.

## Molecular determinants of immune responsiveness in breast cancer and putative role of SLFN11

### Presented by Davide Bedognetti

By exploiting the integrative data available from the cancer genome atlas, we assessed the determinants of immune response in breast cancer (BC) [22]. In that work, we identified that a T helper cell phenotype upregulation is associated with a better prognosis, validating such observation in an independent data set [23]. SLFN11 was discovered in association with thymocyte maturation [1], and appears as an interferon (IFN) regulated gene [5]. To investigate the transcriptional landscape of SLFN11 in BC, we performed a gene expression microarray meta-analysis of more than 7000 cases from 35 publicly available data sets [24]. By pan-transcriptional SLFN11 correlative analysis, we identified 537 transcripts in the top 95th percentile of Pearson’s coefficients with SLFN11. The terms “lymphocyte activation”, “immune response”, and “T cell activation” resulted as top gene ontology enriched processes [25]. We leveraged the method of multiple corresponding analysis, a multivariate statistical process aimed at inferring mutual associations among categorical variables [26]. Thus, we identified a patient cluster defined by elevated SLFN11 expression, ER lack of staining, basal-like PAM50 phenotype, increased CD3D, STAT1 signature [25], and younger age at diagnosis. By penalized maximum likelihood lasso regression [27], we observed a very strong association of SLFN11 with the previously described stroma 1 and stroma 2 signatures [28, 29]. These signatures usually appear upregulated in basal-like BC and in ER- tumors responding to chemotherapy. Finally, using Cox proportional hazard regression, we characterized SLFN11 high levels, high proliferation index, and ER negativity as independent parameters for longer disease-free interval in patients undergoing chemotherapy. Altogether, our data point toward a role for SLFN11 in BC, in likely connection with the immune system modulation in such disease entity.

## SLFN11 and sensitivity to irinotecan in colon cancer

### Presented by Sana Intidhar Labidi-Galy

**Fig. 4:**
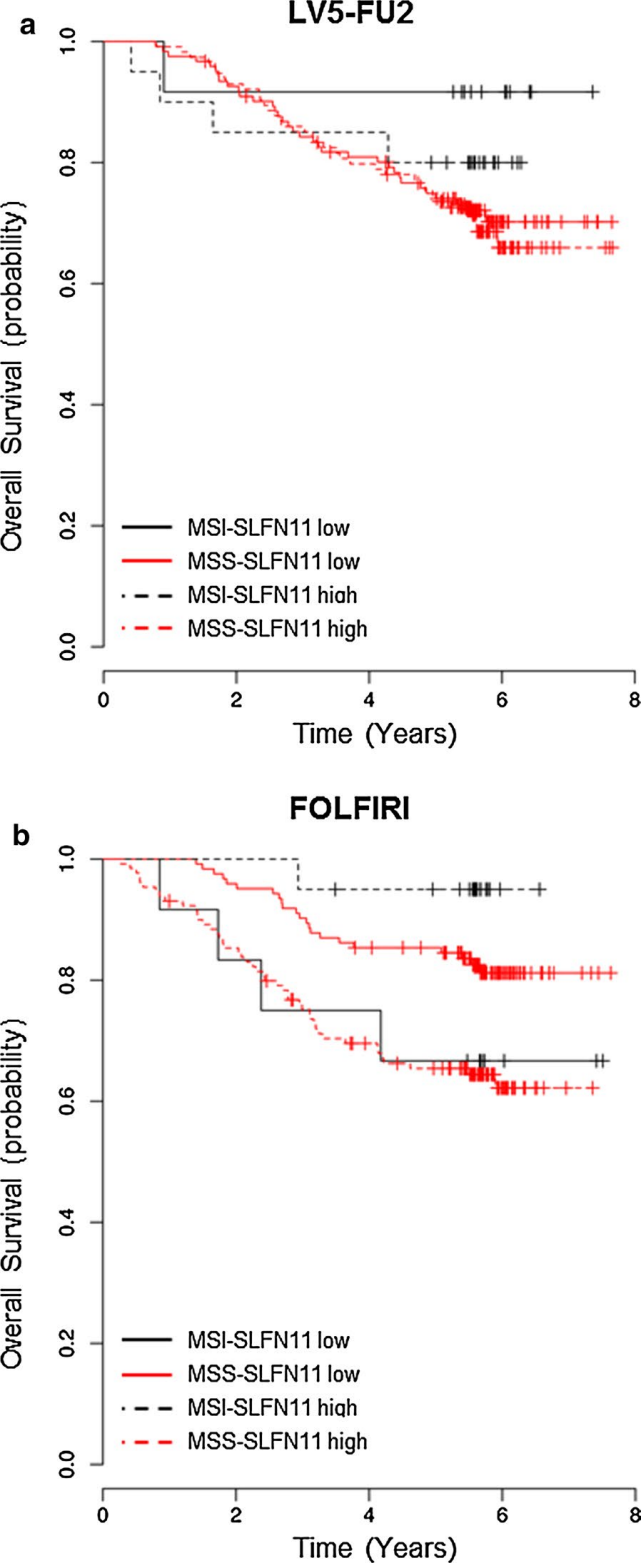
Overall survival in patients of the PETACC3 study according to SLFN11 levels and MSI status. **a** Overall survival in the 268 patients treated with LV5-FU2 regimen. **b** Overall survival in the 285 patients treated with FOLFIRI regimen

SLFN11 has recently been identified as the protein with the highest correlation with sensitivity to topoisomerase I inhibitors such as irinotecan in the NCI60 cancer cell lines [3] and in the cancer cell line encyclopedia [4]. We investigated the correlation between the expression of SLFN11 and survival in colon cancer patients treated in the PETACC3 study, a randomized phase III trial that included 3278 patients in the adjuvant setting and compared two regimens of chemotherapy: half of the patients received LV5-FU2 regimen (5-FU based chemotherapy) while the other half received FOLFIRI regimen (LV5-FU2 and irinotecan). No significant improvement in disease-free survival (DFS) or overall survival (OS) was detected by adding irinotecan to LV5-FU2 as adjuvant therapy [30]. Patients’ tumor samples were collected and gene expression profile analysis was performed on 553 tumors [31]. In the FOLFIRI regimen group (285 patients), we surprisingly observed that patients with SLFN11-high tumors manifested a worse outcome than those having SLFN11-low tumors (7 years-OS 70.6% vs 79.3%, HR = 1.53, 95% CI 1.012–2.503, Log-Rank *p* = 0.044), while in the LV5-FU2 group (268 patients who received only LV5-FU2 regimen) SLFN11 levels did not have any impact on survival (7 years-OS 71.6% vs 73.0%, HR = 1.034, 95% CI 0.667–1.603, *p* = 0.88). We then investigated the interaction between SLFN11 levels and microsatellite instability (MSI) status [32], observing a trend toward increased levels of SLFN11 in MSI-high tumors (40/64 = 62.5%) compared to microsatellite stable (MSS) tumors (244/489 = 49.89%, Fisher test *p* = 0.06). We divided the patients into four groups: group 1 (MSI-high and SLFN11-high), group 2 (MSI-high and SLFN11-low), group 3 (MSS and SLFN11-high) and group 4 (MSS and SLFN11-low). In the LV5-FU2 group, there was absolutely no difference whether tumors were SLFN11-high/low, MSI or MSS tumors (Fig. 4a); in the FOLFIRI group, we observed that among tumors with MSI-high—having a very high rates of mutation [33]—the patients with SLFN11-high tumors showed a better outcome compared to those having MSI-High but SLFN11-low tumors (7 years-OS 95% vs 66.7%, HR = 0.129, 95% CI 0.014–1.156, *p* = 0.067). Inversely, in patients with MSS tumors, we observed a worse outcome in patient SLFN11-high than in SLFN11-low (7 years-OS 64.6% vs 82.1%, HR = 2.348, 95% CI 1.412–3.904, *p* = 0.001) (Fig. 4b). Analyzing these data in a multivariable model, we demonstrated that the interaction between MSI status and SLFN11 was significant (*p* = 0.011). One study addressed the prognostic significance of SLFN11 overexpression in colorectal (CRC) cancers. The cohort included 261 patients with stage II or III CRC cancers treated with oxaliplatin-based adjuvant chemotherapy. SLFN11 was assessed by immunohistochemistry [34]. Overall, CRC with high SLFN11 levels did not show prolonged survival. A substantial benefit from SLFN11 overexpression was observed only in the sub-group of patients with *KRAS* wild-type tumors. SLFN11 overexpression did not have impact on the outcome of patients harboring somatic *KRAS* mutation (exon 2). There is an overlap between MSI and *KRAS* status, with 90% of CRC MSI-high being *KRAS* wild-type (*p* < 0.001) [35]. Together, these observations suggest that a subgroup of CRC tumors MSI-high, *KRAS* wild-type, overexpressing SLFN11, is very likely to benefit from DDA-based adjuvant chemotherapy. In the future, it would be interesting to better identify this sub-group of tumors and investigate at the molecular level the mechanisms underlying such benefit.

## Consensus conclusions

### Shared by all the co-authors

1. SLFN11 is a protein with a causal association with response to DDA in cancer cells.
2. SLFN11 is induced by IFN, but the current relationship between TILs and SLFN11 expression in cancer tissues is not known.
3. SLFN11 can be assessed in human cancer tissues by IHC, with wide range of expression.
4. Several preclinical and clinical models points toward SLFN11 as a predictive marker of response to DDA and PARP inhibitors.
5. SLFN11 expression may be related to mutational burden and MSI in colon cancer.
6. At present, the predictive role of SLFN11 expression in human tumors is unclear and needs further investigation.
7. At present, there is no consensus on the exact function of SLFN11 in health and disease, but all available evidence points toward its relevance in cancer.
